# Supplementary material for: Learning curve and surgical outcome of robotic assisted colorectal surgery with ERAS program
Source: Sci Rep. 2022 Nov 29;12:20566. doi: 10.1038/s41598-022-24665-w (PMC9709162; doi:10.1038/s41598-022-24665-w)
Supplement: Supplementary file 1 — Supplementary Information 1. [file 41598_2022_24665_MOESM1_ESM.zip › LSC Raw data-Ñ[▒K/060/P_ñw╝╨░O▒Kñσ.pdf]

[redacted]

Pathologic diagnosis:
Colon, terminal ileum, and appendix, laparoscopic right hemicolectomy --- Intramucosal adenocarcinoma arising in a tubulovillous adenoma, of ascending colon. Tumor invades to mucosa muscularis. The appendix, ileocecal valve, bilateral cut ends and mesocolic lymph nodes (0/29) are free of tumor.

Prognostic and predictive factor:
1. Tumor size: 4x2.7 cm.
2. Depth of invasion: Intramucosal carcinoma, invasion of muscularis mucosae.
3. Histologic grade: G2: Moderately differentiated.
4. Angiolymphatic invasion: Not identified.
5. Perineural invasion: Not identified.
6. Tumor deposits (discontinuous extramural extension): Not identified.
7. Surgical margins: a) Proximal Margin: Uninvolved by invasive carcinoma, distance of tumor from margin: 9.5 cm. b) Distal Margin: Uninvolved by invasive carcinoma, distance of tumor from margin: 6 cm. c) Radial or Mesenteric Margin: Uninvolved by invasive carcinoma.
8. Lymph node status: Uninvolved (0/29).
9. Extranodal involvement: Not identified.
10. Tumor regression grading S/P CCRT: No known presurgical therapy.
11. Type of polyp in which invasive carcinoma arose: Tubulovillous adenoma.
12. Additional pathologic findings: Not identified.
13. Pathological TNM Stage: pTisN0(According to the eighth edition, 2017, American Joint Committee on Cancer Staging Guidelines for Tumors / 2017 CAP guideline).
14. TNM descriptors: Not applicable.

Gross description:
The specimen consists of a segment of colon, 13 cm in length, and 9.5 cm in proximal circumference, 5.5 cm in distal cut end circumference, and a segment of terminal ileum, 6 cm in length, and 3.6 cm in cut end circumference. An unremarkable appendix, 4.4x0.6 cm, is noted. A gray tan polypoid tumor, 4x2.7 cm, is noted, 6 cm to the nearest cut end. On cross section, no definite stromal invasion is identified grossly. The appendix, ileocecal valve, and both cut ends are free of tumor. Representative part for section: A) proximal cut end B) distal cut end C) ileocecal valve D) appendix E-H) tumor J-L) lymph nodes.

Note:
This case has been peer reviewed by two doctors.
#T-59420_2 #M-82632_2 9 1105 00000F
#T-C4520_2 #M-09183_2 0 1105 000000
#T-59200_2 #M-09183_2 0 1105 000000

已電子簽章

敬告：本單僅供醫師參考使用，不具任何申請效力！！
請病患妥為保管，若不慎遺失需自負個人資料外洩責任！
